# Cancer-derived EVs show tropism for tissues at early stage of neoplastic transformation

**DOI:** 10.7150/ntno.47226

**Published:** 2021-01-01

**Authors:** Mariangela Garofalo, Alessandro Villa, Electra Brunialti, Daniela Crescenti, Giulia Dell'Omo, Lukasz Kuryk, Andrea Vingiani, Vincenzo Mazzaferro, Paolo Ciana

**Affiliations:** 1 Department of Health Sciences, Center of Excellence on Neurodegenerative Diseases, University of Milan, Italy.; 2 Current address: Department of Pharmaceutical and Pharmacological Sciences, University of Padova, Italy.; 3 Department of Pharmacological and Biomolecular Sciences, University of Milan, Milan, Italy.; 4 Targovax Oy, Clinical Science, Helsinki, Finland.; 5 National Institute of Public Health - National Institute of Hygiene, Department of Virology, Warsaw, Poland.; 6 Department of Oncology and Hemato-Oncology, University of Milan, Milan, Italy.; 7 Istituto Nazionale Tumori Fondazione IRCCS, National Cancer Institute, Milan, Italy.

**Keywords:** Extracellular vesicles, oncolytic viruses, early-stage cancer therapy, drug delivery, *in vivo* imaging

## Abstract

From the past decade, extracellular vesicles (EVs) have attracted considerable attention as tools for the selective delivery of anti-neoplastic drugs to cancer tissues. Compared to other nanoparticles, EVs display interesting unique features including immune compatibility, low toxicity and the ability to encapsulate a large variety of small- and macro-molecules. However, in virtually all studies, investigations on EVs have been focused on fully transformed cancers: the possibility to apply EV technology also to early-stage tumors has never been explored.

**Methods:** Herein, we studied the ability of cancer-derived EVs to recognize and deliver their cargo also to incipient cancers. To this purpose, EV biodistribution was studied in MMTV-NeuT genetically modified mice during early mammary transformation, in fully developed breast tumors and in the normal gland of wild type syngeneic mice. EVs were loaded with indocyanine green (ICG), a near-infrared (NIR) dye together with oncolytic viruses and i.v. injected in mice. The nanoparticle biodistribution was assayed by *in vivo* and *ex vivo* optical imaging (detecting the ICG) and semiquantitative real-time PCR (measuring the adenoviral genome) in different tissues.

**Results:** Our results demonstrate the ability of cancer-derived EVs to recognize early-stage neoplastic tissues opening the possibility to selectively deliver theranostics also for tumor prevention.

**Conclusions:** Taken together our study demonstrates the ability of EVs to recognize and deliver diagnostic and therapeutic agents not only to fully transformed tissues but also to early stage tumors. These findings pave the way for the synthesis of “universal” EVs-based formulation for targeted cancer therapy.

## Introduction

In the recent years, EVs, nanometer- to micron-sized lipid bilayered vesicles able to shuttle biological molecules over long distances 1 and have gained considerable attention as emerging tools for the delivery of theranostics agents 2-7. EVs physiologically encapsulate metabolites, proteins or miRNA and have been artificially loaded with small molecule and biologics as therapeutic or diagnostic cargos, with the aim of obtaining their targeted delivery to specific cell populations. EVs encapsulation can improve drug pharmacokinetics providing a tissue-specific homing, protecting the delivered molecules from degradation and their early systemic release before reaching the target tissue 8-10. Recent works provided compelling evidence of the tumor-selective homing of cancer-derived EVs 11-13; this tropism is not specific for the tumor tissue originating the EVs, but it is maintained for different cancer types arising even from different species that might reflect an ancillary conserved mechanism of intercellular communication operating during tumor progression 14. For these features, EVs are particularly attractive for the delivery of anti-neoplastic drugs, including small molecules and biologics 15, because they can direct the therapy selectively to the tumor site: a formulation-mediated targeted therapy with the potential to increase the local concentration of EVs-encapsulated drugs, while reducing systemic side effects. A current limitation of this formulation is that can be applied only for the treatment of advanced cancers, because demonstration of the homing capability of EVs for early cancer stage has not been provided yet. The possibility to deliver high dosages of diagnostics, chemo-preventive agents, chemotherapies or biopharmaceuticals locally, to the site of initial neoplastic transformation is an attractive strategy that is expected to exploit the anticancer effects of drugs on the emerging tumor with potentially relevant consequences on patients' survival. Hence, in the current study, we set to investigate whether the systemic administration of cancer derived EVs is able to deliver theranostics to early stage mammary cancers generated in a genetic mouse model of breast cancer (MMTV-NeuT mice) 16. For these experiments, we encapsulated into EVs a diagnostic fluorescent dye 17 and an oncolytic virus 18-21 as tracking agents for the characterization of the whole-body biodistribution of the formulations. Herein, we show that cancer-derived EVs are able to target the mammary glands in MMTV-NeuT mice as soon as they start developing a neoplastic tissue, thus suggesting the possibility to deliver theranostic cargos of EVs also to early stage neoplasia.

## Materials and Methods

### Cell lines and virus

MC-38 mouse colon cancer and 4T1 mouse mammary tumor cell lines were purchased from the American Type Culture Collection (ATCC, USA). Cells were cultured at 37 °C and 5 % CO_2_ in Dulbecco's modified eagle medium (DMEM, Lonza, Switzerland) supplemented with 10% fetal bovine serum (FBS, Gibco Laboratories, USA), 1 % of 100 u/mL penicillin/streptomycin (Gibco Laboratories) and 1% L-glutamine (Gibco Laboratories). Oncolytic adenovirus Ad5D24 has been characterized by performing titration (VP/ml) and molecular analyses (PCR, restriction enzyme assay) to check virus genome stability and integrity as described elsewhere 22, expanded in human lung cancer cell line A549 and purified on cesium chloride gradients 23. The viral particle concentration was determined by OD_260_-reading and standard TCID_50_ (tissue culture infectious dose 50) assay was performed to determine infectious particle titer.

### Production of EV, EV-virus and ICG loaded EV formulations

In order to produce EVs, 2.6 x 10^6^ MC-38 cells were plated into T-175 flask in medium supplemented with 5 % FBS. The FBS growth media was ultra-centrifuged overnight (110 000 x g at 4°C for 18 hours, Optima LE-80K ultracentrifuge, rotor type 50.2, Beckman Coulter) to remove EVs present in serum.

EVs were isolated from the conditioned medium using differential centrifugation steps as previously reported 9,11,12. First the conditioned medium was centrifuged at 500 × g in 4°C for 10 minutes to pellet cells (Allegra X-15R Centrifuge, Beckman Coulter). Then, the supernatant was collected and ultra-centrifuged for 2 hours at 100 000 × g in 4°C, using Optima L-80 XP ultra-centrifuge (Beckman Coulter) with rotor SW32Ti (Beckman Coulter). The supernatant was aspirated and EV- containing pellets containing re-suspended in PBS (Lonza) 100 μL and stored at - 80 °C.

EV-encapsulated virus (EV-Virus) were produced as previously described, Virus samples were incubated in 100 mM NaOH at room temperature for 20 minutes in order to inactivate any free not EV encapsulated virus present. Free virus used as control was always inactivated for each experiment performed as previously reported 9. Samples were subsequently neutralized by the addition of HCl 0.1 M.

EVs and EV-Virus were loaded with Indocyanine green (EV-ICG) and prepared by incubating 1×10^8^ - 5×10^9^ EVs in PBS for 12 h at 4°C with 10ug/mL ICG (Sigma). Next, the samples were centrifuged at 150 000 x g for 3 h to pellet the EVs. The supernatant containing unbound ICG was removed, and the EV-pellet was washed by suspending it in PBS and pelleting it again at 150 000 × g. The final EV-ICG-Virus pellet were re-suspended in 100 μL of PBS and stored at -80 °C until use. EV-formulations have been further characterized as previously reported 9,11,12,14.

### Quantitative real-time PCR

qPCR for adenovirus E4 copy number was carried out according to the protocol previously described 24 primer forward: 5'-GGA GTG CGC CGA GAC AAC-3', primer reverse: 5'-ACT ACG TCC GGC GTT CCA T-3', probe E4: 5'-(6FAM)-TGG CAT GAC ACT ACG ACC AAC ACG ATC T- (TAMRA)-3'. Total DNA was extracted from resected brains, tumors, livers, blood from C57BL/6 mouse model after 24h post treatment, using the QIAamp DNA Blood Mini Kit (Qiagen, Hilden, Germany) according to manufacturer's protocol. Subsequently isolated DNA was analyzed for adenoviral E4 copy number normalized to murine beta-actin (liver, blood): primer forward: 5'-CGA GCG GTT CCG ATG C-3', primer reverse: 5'-TGG ATG CCA CAG GAT TCC AT-3', probe murine beta-actin: 5'-(6FAM)-AGG CTC TTT TCC AGC CTT CCT TCT TGG-(TAMRA)-3'. Samples were analyzed using LighCycler qPCR machine (LighCycler 480, Roche, Basel, Switzerland).

### *In vivo* experiments and pharmacological treatments

All animal experimentations were performed and approved by the Italian Ministry of Research and University and controlled by a Departmental panel of experts (permission numbers: 12-12-30012012, 547/2015, 214/2020). MMTV-NeuT and C57Bl/6 mice were used for the experiments. The acclimatization period was 14 days prior MC-38 and 4T1 cancer cell injections in C57Bl/6 mice, health status of the engrafted mice was monitored daily and as soon as signs of pain or distress were evident, they were euthanized. For early cancer detection, female MMTV-NeuT mice were enrolled in the experiments at week 6 of age, when hyperplasia was evident and full transformation of the breast has not yet occurred 25. EV-ICG-Virus-MC38 (n=5) (1×10^8^ particles/injection + 1×10^8^ viral particles/injection +10 ug/mL) were administered i.v in a volume of 100 µl.

### *In vivo* and *ex vivo* imaging

*In vivo* and *ex vivo* fluorescence imaging has been carried out 24 hours post EV treatments using IVIS Lumina II Quantitative Fluorescent Imaging (PerkinElmer, Waltham, MA, USA) with suitable filters (Cy5.5) and following the manufacturer's instructions for fluorescence background subtraction. Mice were anaesthetized using Isofluorane (Isofluorane-Vet; Merial, Lyon, France) and kept under anesthesia during imaging sessions carried out with the Imaging System (5 min for dorsal view and 5 min for ventral view) (IVIS Lumina II Quantitative Fluorescent and Bioluminescent Imaging; PerkinElmer, Waltham, MA, USA). Photon emission in selected body areas was measured using the Living Image Software 3.2 (PerkinElmer). For the *ex vivo* imaging, mice were treated with luciferin 15 min prior euthanasia by cervical dislocation and *ex vivo* imaging of the selected organs were carried out immediately after death. The quantification was done with Living Image Software 3.2 (PerkinElmer).

### Histopathological analysis

In order to perform histopathological evaluation, breast and tumor tissues explanted from MMTV-NeuT mice were immersed in a 4% formaldehyde solution for 24 hours in order to obtain chemical fixation. Samples were then dehydrated with ethanol, washed with xylene and included in paraffine; then they were cut into 4 and 10 µm thick sections for hematoxylin/eosin staining and fluorescence imaging, respectively. Slides were then analyzed with a confocal microscope (Nikon A1R laser scanning confocal microscope) to acquire the fluorescent signal released by ICG and compared with the adjacent hematoxylin/eosin sections acquired with a Virtual Slide Microscope (Olympus VS120). Throughout the process of fixing and paraffining, the samples were kept out of the light, in order to avoid the loss of the fluorescent signal. Based on our previous experiments carried out with a similar tumor model and *in vivo* imaging endpoints 11,12,14 and taken into account the experimental error, we calculated that five mice per experimental group were sufficient to assess the homing capabilities of EV-formulations in mice bearing hyperplastic or transformed mammary gland *versus* syngeneic healthy controls. Sampling and histology on the resected tumor specimens were determined by professional pathologists (at Pathology Department, Istituto Nazionale Tumori IRCCS Foundation, Milan, Italy). They were not blinded when assessing EV homing with *in vivo* imaging, while were blinded when assessing EV homing in histopathological samples. Statistical significance was assessed by using one-way ANOVA with Tukey's Multiple Comparison test and nonparametric Mann-Whitney test. All statistical analysis, calculations and tests were performed using GraphPad Prism 5 (GraphPad Software, San Diego, CA).

## Results and Discussion

**Figure 1:**
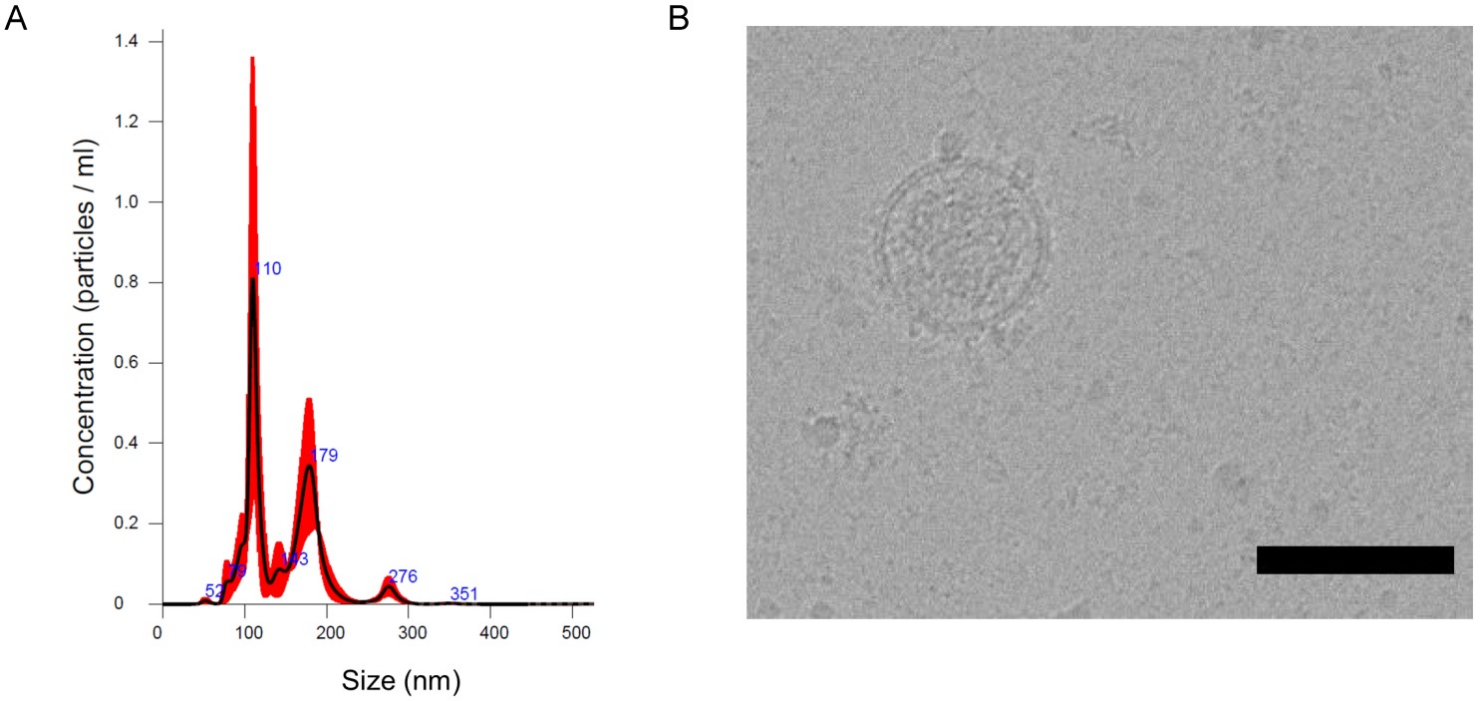
** Characterization of cancer derived EV-formulations (EV-ICG-Virus-MC-38).** (A) Representative particle size distribution analysis of plasma-derived EVs obtained by nanoparticle tracking analysis (NTA). (B) EV imaging by cryo-electron microscopy, scale bar: 100 nm.

**Figure 2:**
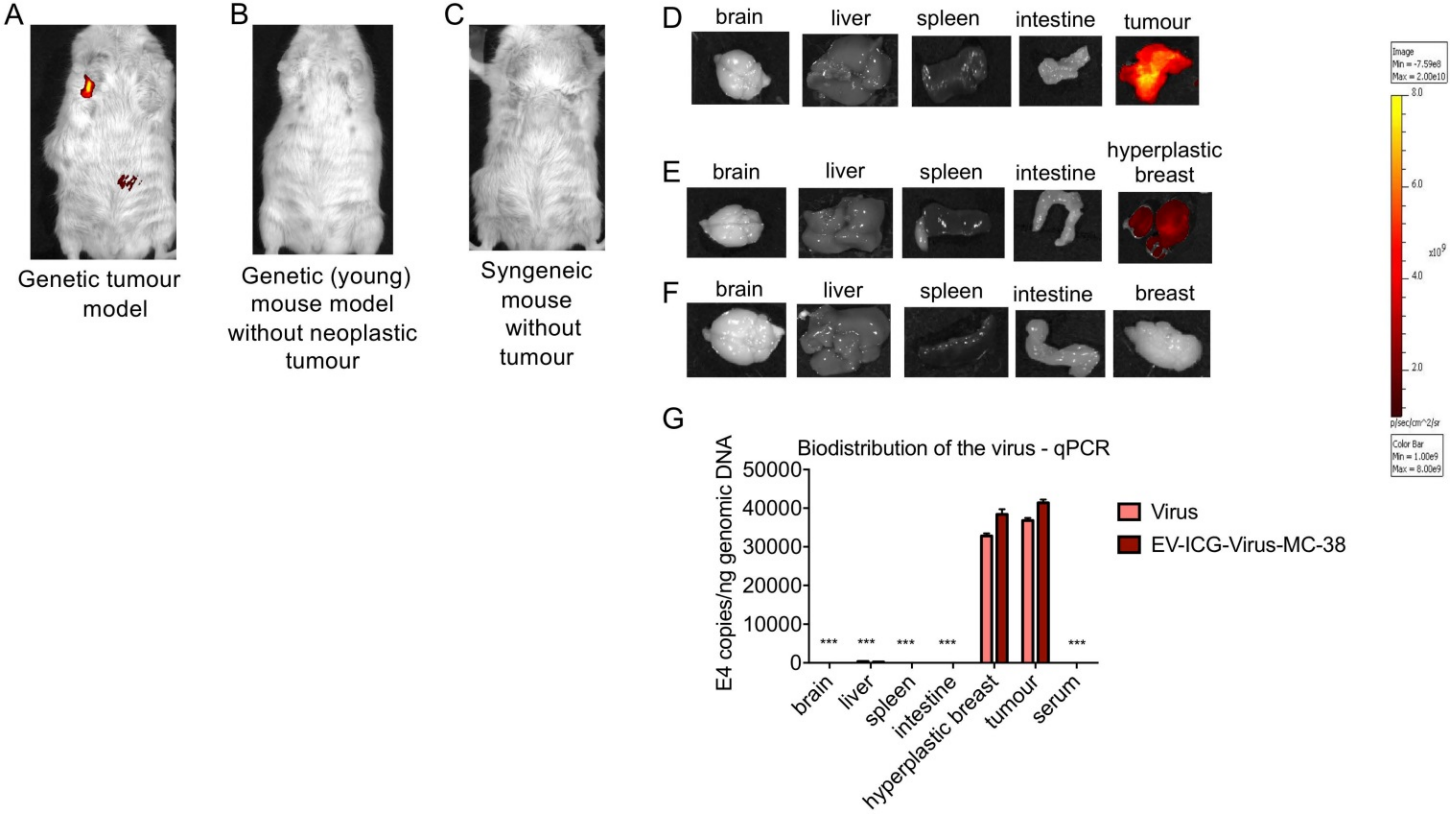
** Cancer derived EV-formulations loaded with diagnostic and theranostics agents are able to target the tumor site and early phase neoplastic transformation.** (A-C) Representative images of the whole-body photon emission (fluorescence) in MMTV-NeuT and syngeneic mice i.v treated with EV-ICG-Virus-MC-38. (D-F) Representative images of the photon emission in organs explanted from MMTV-NeuT and syngeneic mice. (G) Adenoviral copies towards E4 gene were measured by qPCR from euthanized mice's organs at the end of the treatment. Error bars mean+/- SD *p<0.05, **p<0.01, ***p<0.001.

**Figure 3:**
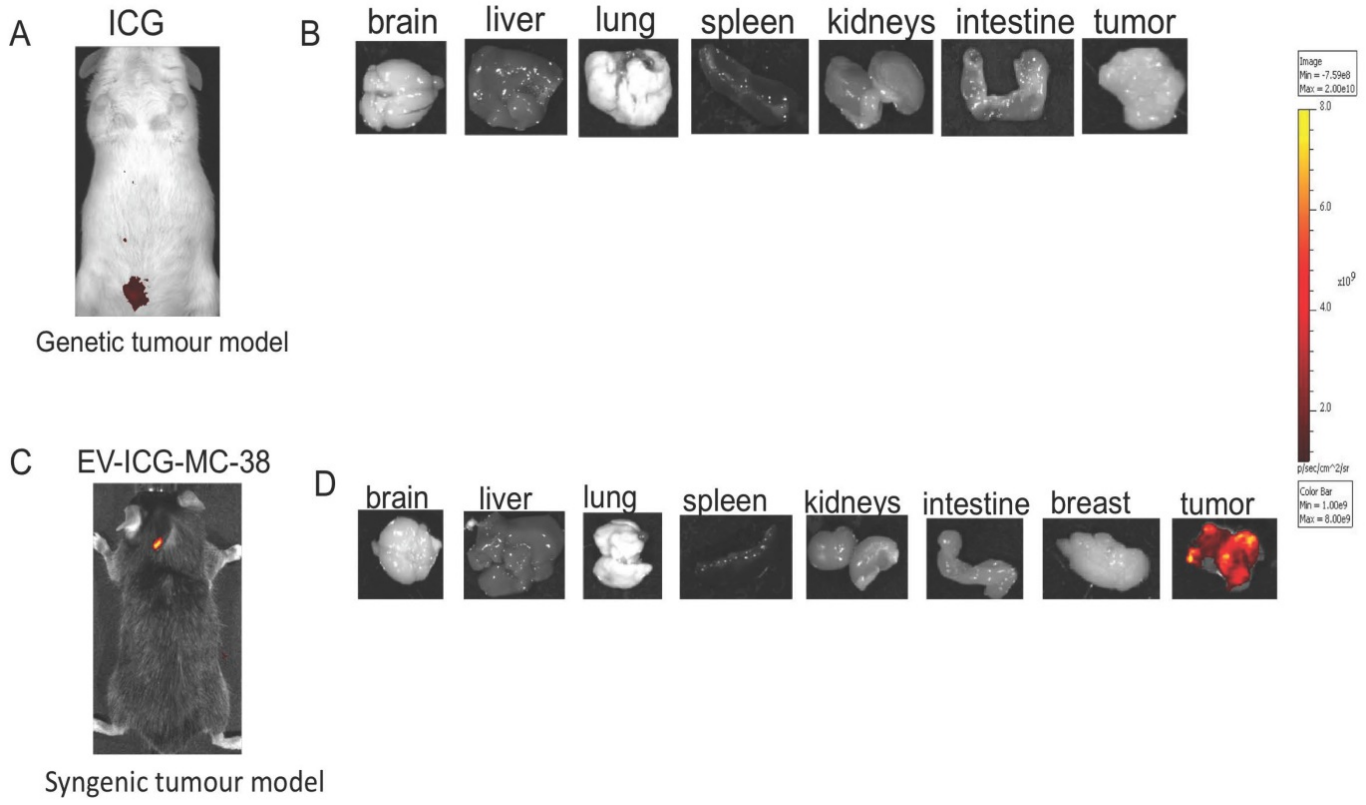
** Indocyanine green not loaded into EV-formulations cannot target the tumor site.** (A-B) Representative images of the photon emission (fluorescence) *in vivo* and in organs explanted from MMTV-NeuT mice i.v. treated with ICG. (C-D) Representative images of the photon emission (fluorescence) in the tumor area and in organs explanted from syngeneic mice i.v. treated with EV-ICG-MC-38.

**Figure 4:**
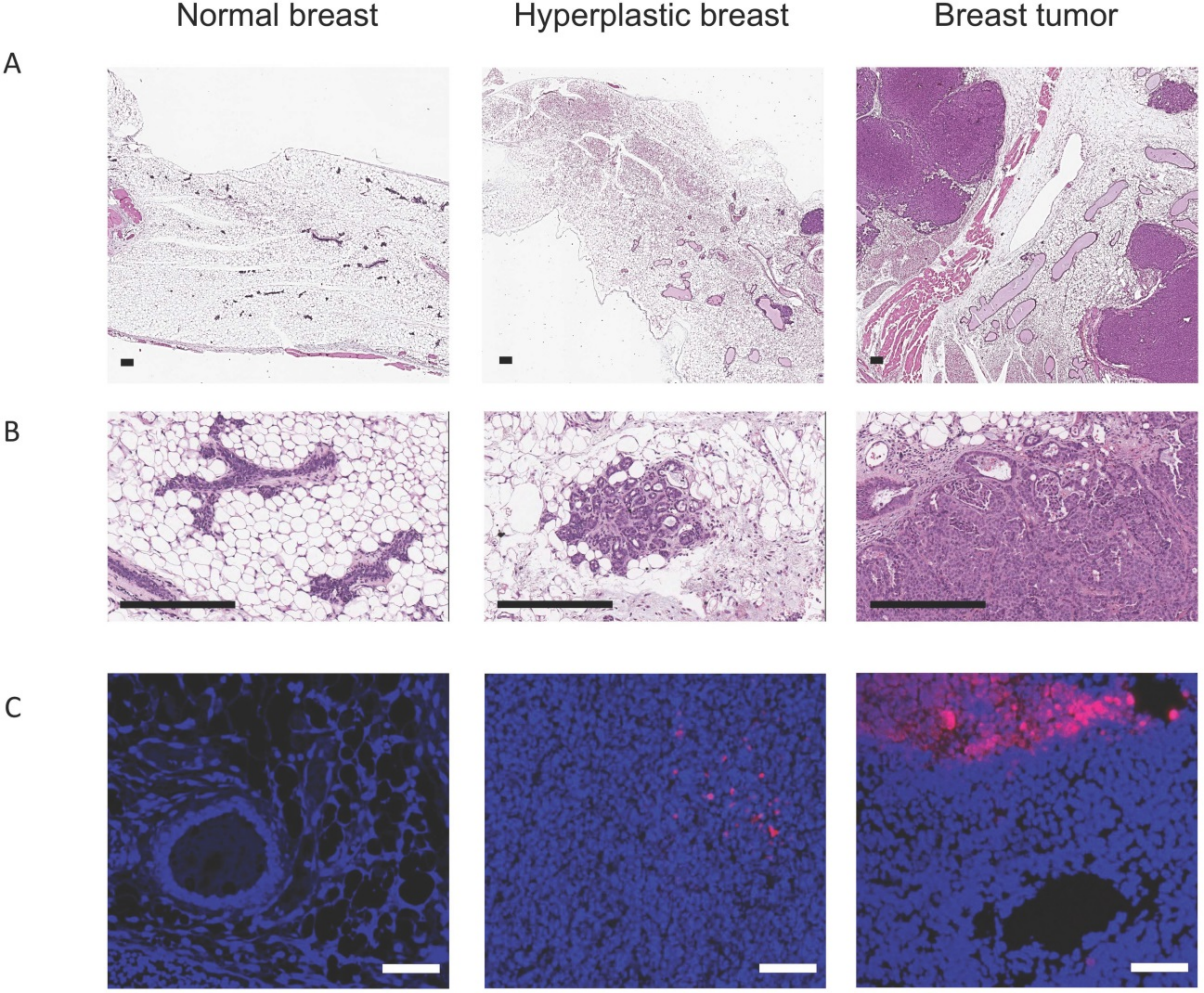
** Cancer derived EVs target neoplastic lesions.** (A-B) Histopathological examination on normal breast, hyperplastic breast and breast tumour has been performed using Haematoxylin and Eosin staining (Scale bar: 2 mm). (C) Analysis of normal breast, hyperplastic breast and breast tumour by confocal microscopy. Nuclei stained with DAPI (blue), ICG signal in purple (Scale bar: 50 µm).

The recent work from our and other laboratories showed that tumor-derived EVs can be used to deliver diagnostics or therapeutics agents selectively to fully transformed tissues 12,14,9-27, while it was not clear the extent to which the tumor-tropism was applicable also to early-stage cancers. Herein, we studied the EV biodistribution in the MMTV-NeuT transgenic mouse, a genetic model of breast cancer 25, in which transformation of the mammary glands slowly progress from microscopic lesions detectable at weeks 6-8, to *in situ* neoplasia at weeks 9-15 until the appearance of a fully transformed invasive tumor usually arising after week 15 16. EVs from the MC-38 cancer cell line were prepared as previously described and displayed biophysical characteristics, in terms of size, distribution, and Zeta-potential, analogous to those produced in our previous work 14 (Figure 1A); as an additional test, cryo-EM experiments confirmed the EV size, morphology and integrity (Figure 1B). In order to track the destiny of cancer derived EV-Virus formulations within the body, we have loaded the biocompatible nanoparticles with ICG together with an oncolytic adenovirus (OA). The biodistribution was measured by *in vivo* and *ex vivo* fluorescence imaging and by semiquantitative real-time PCR to measure the amount of adenoviral genome in different tissues. MMTV-NeuT mice at 6 and 24 weeks of age and syngeneic controls were i.v. injected with the EV-ICG-Virus-MC38 formulations and 24 h post-treatment fluorescent emission was acquired by *in vivo* (Figure 2A,B,C) and *ex vivo* imaging (Figure 2D-F). Results showed that fluorescence emission originated mainly from the hyperplastic mammary gland (Figure 2A,D) or, to a greater extent, from the transformed breast of the MMTV-NeuT mice (Figure 2B,E), but not from the normal gland (Figure 2C,F). The qPCR analysis confirmed this conclusion showing that the OA content of the vesicles was delivered preferentially to the hyperplastic/neoplastic tissue of the MMTV-NeuT mice (Figure 2G). Interestingly, the tumor-specific tropism could not be detected when ICG was administered alone (Figure 3A-B) confirming that the fluorescence accumulation was due to the EV-mediated delivery of the dye. Furthermore, the EV-biodistribution in a syngeneic mouse model, subcutaneously injected with 4T1 murine breast cancer cells, showed a selective homing of EV-particles into the neoplastic tissue without targeting the mammary gland (Figure 3C-D). The preferential accumulation of the dye in the transformed breast was confirmed by confocal microscopy analysis performed on normal, hyperplastic and transformed breasts isolated from MMTV-NeuT and syngeneic mice (Figure 4). During the transition phase from pre-invasive to invasive ductal breast carcinoma, both mammary glands and their surrounding tissue experience extensive changes due to secretion of inflammatory factors by tumor cells, cancer-associated fibroblasts and macrophages. Such changes include a re-organization of the extracellular matrix and modifications in gene and protein expression profiles. Remarkably, tumoral EVs did recognize the pathological mammary glands independently from the stage: this observation suggests that the recognition mechanism driving the specific tropism of tumoral EVs could reflect these early changes occurring in the transforming tissue. Alternatively, it cannot be excluded that tumoral EVs present a repertoire of sensors allowing the migration towards tumoral microenvironments independently from the stage. Being the mechanism behind EV tropism still largely unknown, the reported results could offer a valuable hint for the identification of molecular determinants involved in the target recognition that should be conserved in both hyperplastic and fully transformed cells. Certainly, a thorough characterization of tumor-derived EVs is needed to fully exploit the great potential of these nanoparticles for diagnostic and therapeutic purposes.

Whatever is the mechanism underlying the tumor selectivity, our data provide the rationale for novel potential clinical and diagnostical applications of EVs in the management of patients carrying early-stage cancers. We have previously demonstrated that the EV encapsulation prevents chemotherapeutic treatments to induce systemic inflammatory reactions, suggesting that EVs are able to deliver cytotoxic drugs to tumoral tissues to a sufficient amount for reaching therapeutic effects 9,12 and with a sufficient selectivity to avoid damages to other tissues 12. Since a local high concentration of cytotoxic agents might be reached in this condition, our study pave the way for possible applications of chemotherapy in preventive clinical protocols aimed at the eradication of the disease before its full progression. Moreover, since EVs can be loaded with MRI or PET contrast agents 28,29, their high local concentration might boost the sensitivity of tumor detection, thus influencing the clinical management of patients carrying early-stage cancers.

Despite these considerations, EV-mediated diagnostic and therapeutic applications are still in their infancy: a more comprehensive understanding of the role of EVs both in the progression of early and fully transformed cancer types is needed. Several issues need to be addressed before EVs use can be translated from the bench to clinical applications. Firstly, the methods for obtaining pure and intact EVs for routing clinical use need the application of reproducible isolation protocols that guarantee scale-up of sample volume, purity, speed, yield, and tumor specificity; finally, the clinical use of cancer-derived EVs certainly requires the clear definition of their possible side effects due to the potentially harmful cargo they are delivering together with the theranostic molecules.

## Conclusions

Taken together the results of our study demonstrate the ability of EVs to recognize and deliver their diagnostic and therapeutic cargos not only to fully transformed tissues 11,12, but also to early stage tumors. Since chemotherapeutic drugs 30,31 as well as macromolecules or entire virus 9,32 and diagnostic agents for cancer imaging 28,29 can be encapsulated inside EVs, it is possible to envisage several applications in early-stage cancer diagnosis and therapy: high local concentration of PET or MRI contrast agents 33 might boost detection sensitivity while locally increasing the concentration of chemotherapy, have the potential to increase efficacy and greatly reduce side effects 12. Still, some technical issue needs to be addressed for a reproducible and safe use of these tools including a standard, clinical grade purification protocol. The knowledge of the molecular determinants underlying their cancer-specific tropism will certainly facilitate their clinical translation by setting better purification protocols and perhaps in the future allowing the synthesis of “universal” EVs-based formulation for targeted cancer therapy.

## Abbreviations

EVs: Extracellular Vesicles
MMTV-NeuT mice: genetic mouse model of breast cancer
ICG: Indocyanine green
EV-Virus: EV-encapsulated virus
EV-ICG: EV-encapsulated ICG
OA: oncolytic adenovirus

## References

[1] Yáñez-Mó M, Siljander PRM, Andreu Z, Zavec AB, Borràs FE, Buzas EI (2015). Biological properties of extracellular vesicles and their physiological functions. *J Extracell Vesicles*.

[2] Gangadaran P, Li XJ, Kalimuthu S kumar, Min OJ, Hong CM, Rajendran RL (2018). New Optical Imaging Reporter-labeled Anaplastic Thyroid Cancer-Derived Extracellular Vesicles as a Platform for *In vivo* Tumor Targeting in a Mouse Model. *Sci Rep*.

[3] Jang SC, Kim OY, Yoon CM, Choi DS, Roh TY, Park J (2013). Bioinspired exosome-mimetic nanovesicles for targeted delivery of chemotherapeutics to malignant tumors. *ACS Nano*.

[4] Jiang XC, Gao JQ (2017). Exosomes as novel bio-carriers for gene and drug delivery. *Int J Pharm*.

[5] Kooijmans SAA, Vader P, van Dommelen SM, van Solinge WW, Schiffelers RM (2012). Exosome mimetics: A novel class of drug delivery systems. *Int J Nanomedicine*.

[6] El Andaloussi S, Mäger I, Breakefield XO, Wood MJA (2013). Extracellular vesicles: Biology and emerging therapeutic opportunities. *Nat Rev Drug Discov*.

[7] van der Meel R, Fens MHAM, Vader P, van Solinge WW, Eniola-Adefeso O, Schiffelers RM (2014). Extracellular vesicles as drug delivery systems: Lessons from the liposome field. *J Control Release*.

[8] Fais S, O'Driscoll L, Borras FE, Buzas E, Camussi G, Cappello F (2016). Evidence-Based Clinical Use of Nanoscale Extracellular Vesicles in Nanomedicine. *ACS Nano*.

[9] Garofalo M, Saari H, Somersalo P, Crescenti D, Kuryk L, Aksela L (2018). Antitumor effect of oncolytic virus and paclitaxel encapsulated in extracellular vesicles for lung cancer treatment. *J Control Release*.

[10] Lener T, Gimona M, Aigner L, Börger V, Buzas E, Camussi G (2015). Applying extracellular vesicles based therapeutics in clinical trials - An ISEV position paper. *J Extracell Vesicles*.

[11] Garofalo M, Villa A, Rizzi N, Kuryk L, Mazzaferro V, Ciana P (2018). Systemic Administration and Targeted Delivery of Immunogenic Oncolytic Adenovirus Encapsulated in Extracellular Vesicles for Cancer Therapies. *Viruses*.

[12] Garofalo M, Villa A, Rizzi N, Kuryk L, Rinner B, Cerullo V (2019). Extracellular vesicles enhance the targeted delivery of immunogenic oncolytic adenovirus and paclitaxel in immunocompetent mice. *J Control Release*.

[13] Hoshino A, Costa-Silva B, Shen TL, Rodrigues G, Hashimoto A, Tesic Mark M (2015). Tumour exosome integrins determine organotropic metastasis. *Nature*.

[14] Garofalo M, Villa A, Crescenti D, Marzagalli M, Kuryk L, Limonta P Theranostics Heterologous and cross-species tropism of cancer- derived extracellular vesicles. 2019; 9.

[15] Watson DC, Bayik D, Srivatsan A, Bergamaschi C, Valentin A, Niu G (2016). Efficient production and enhanced tumor delivery of engineered extracellular vesicles. *Biomaterials*.

[16] Guy CT, Webster MA, Schaller M, Parsons TJ, Cardiff RD, Muller WJ (1992). Expression of the neu protooncogene in the mammary epithelium of transgenic mice induces metastatic disease. *Proc Natl Acad Sci U S A*.

[17] Wang H, Li X, Tse BWC, Yang H, Thorling CA, Liu Y (2018). Indocyanine green-incorporating nanoparticles for cancer theranostics. *Theranostics*.

[18] Capasso C, Magarkar A, Cervera-Carrascon V, Fusciello M, Feola S, Muller M (2017). A novel in silico framework to improve MHC-I epitopes and break the tolerance to melanoma. *Oncoimmunology*.

[19] Kuryk L, Møller A-SW, Garofalo M, Cerullo V, Pesonen S, Alemany R (2018). Anti-tumor specific T-cell responses induced by oncolytic adenovirus ONCOS-102 in peritoneal mesothelioma mouse model. *J Med Virol*.

[20] Cerullo V, Vähä-Koskela M, Hemminki A (2012). Oncolytic adenoviruses: A potent form of tumor immunovirotherapy. *Oncoimmunology*.

[21] Kuryk L, Møller A-SW, Jaderberg M (2019). Abscopal effect when combining oncolytic adenovirus and checkpoint inhibitor in a humanized NOG mouse model of melanoma. *J Med Virol*.

[22] Farzad L, Cerullo V, Yagyu S, Bertin T, Hemminki A, Rooney C (2014). Combinatorial treatment with oncolytic adenovirus and helper-dependent adenovirus augments adenoviral cancer gene therapy. *Mol Ther - Oncolytics*.

[23] Kuryk L, Møller A-S, Vuolanto A, Pesonen S, Garofalo M, Cerullo V (2019). Optimization of Early Steps in Oncolytic Adenovirus ONCOS-401 Production in T-175 and HYPERFlasks. *Int J Mol Sci*.

[24] Koski A, Kangasniemi L, Escutenaire S, Pesonen S, Cerullo V, Diaconu I (2010). Treatment of cancer patients with a serotype 5/3 chimeric oncolytic adenovirus expressing GMCSF. *Mol Ther*.

[25] Boggio BK, Nicoletti G, Carlo E Di, Cavallo F, Landuzzi L, Melani C Interleukin 12-mediated Prevention of Spontaneous Mammary Adenocarcinomas in Two Lines of Her-2/. 1998; 188.

[26] van den Boorn JG, Schlee M, Coch C, Hartmann G (2011). SiRNA delivery with exosome nanoparticles. *Nat Biotechnol*.

[27] Tian Y, Li S, Song J, Ji T, Zhu M, Anderson GJ (2014). A doxorubicin delivery platform using engineered natural membrane vesicle exosomes for targeted tumor therapy. *Biomaterials*.

[28] Varga Z, Gyurkó I, Pálóczi K, Buzás EI, Horváth I, Hegedűs N (2016). Radiolabeling of Extracellular Vesicles with ^99m^ Tc for Quantitative *In vivo* Imaging Studies. *Cancer Biother Radiopharm*.

[29] Shi S, Li T, Wen X, Wu SY, Xiong C, Zhao J (2019). Copper-64 Labeled PEGylated Exosomes for *In vivo* Positron Emission Tomography and Enhanced Tumor Retention. *Bioconjug Chem*.

[30] Jang SC, Kim OY, Yoon CM, Choi D-S, Roh T-Y, Park J (2013). Bioinspired Exosome-Mimetic Nanovesicles for Targeted Delivery of Chemotherapeutics to Malignant Tumors. *ACS Nano*.

[31] Vader P, Mol EA, Pasterkamp G, Schiffelers RM (2016). Extracellular vesicles for drug delivery. *Adv Drug Deliv Rev*.

[32] Ran L, Tan X, Li Y, Zhang H, Ma R, Ji T (2016). Delivery of oncolytic adenovirus into the nucleus of tumorigenic cells by tumor microparticles for virotherapy. *Biomaterials*.

[33] Gangadaran P, Hong CM, Ahn B-C (2018). An Update on *in vivo* Imaging of Extracellular Vesicles as Drug Delivery Vehicles. *Front Pharmacol*.

